# Three Boundary Conditions for Computing the Fixed-Point Property in Binary Mixture Data

**DOI:** 10.1371/journal.pone.0167377

**Published:** 2016-11-28

**Authors:** Leendert van Maanen, Joaquina Couto, Mael Lebreton

**Affiliations:** 1 Department of Psychology, University of Amsterdam, Amsterdam, The Netherlands; 2 Amsterdam Brain and Cognition (ABC), University of Amsterdam, Amsterdam, The Netherlands; 3 Amsterdam School of Economics (ASE), Faculty of Economics and Business (FEB), University of Amsterdam, Amsterdam, The Netherlands; Brain and Spine Institute (ICM), FRANCE

## Abstract

The notion of “mixtures” has become pervasive in behavioral and cognitive sciences, due to the success of dual-process theories of cognition. However, providing support for such dual-process theories is not trivial, as it crucially requires properties in the data that are specific to mixture of cognitive processes. In theory, one such property could be the fixed-point property of binary mixture data, applied–for instance- to response times. In that case, the fixed-point property entails that response time distributions obtained in an experiment in which the mixture proportion is manipulated would have a common density point. In the current article, we discuss the application of the fixed-point property and identify three boundary conditions under which the fixed-point property will not be interpretable. In Boundary condition 1, a finding in support of the fixed-point will be mute because of a lack of difference between conditions. Boundary condition 2 refers to the case in which the extreme conditions are so different that a mixture may display bimodality. In this case, a mixture hypothesis is clearly supported, yet the fixed-point may not be found. In Boundary condition 3 the fixed-point may also not be present, yet a mixture might still exist but is occluded due to additional changes in behavior. Finding the fixed-property provides strong support for a dual-process account, yet the boundary conditions that we identify should be considered before making inferences about underlying psychological processes.

## Introduction

The notion of “mixtures” has become pervasive in behavioral and cognitive sciences. From psychology to economics or reinforcement learning theories, behavior and decisions are now conceptualized as the joint outputs (*mixture*) of multiple cognitive modules. These modules are typically referred to as selves [1], systems [2], and/or computational modules [3–5]. Most theories dissociate two main modules, which lead to the notion of dual-processes theories [6,7]. In this framework, behavioral differences between individuals, and/or behavioral modulations within one individual, are typically attributed to differences in–or modulations of- the relative contributions of the two processes to the behavioral output, i.e. to the *mixture proportion*.

However, despite this strong theoretical stance, whether dual processes jointly account for the final behavior in a given task or context, and whether people can switch between two processes depending on the conditions remains an open research question [8–11]. A crucial reason is that, often, the only behavioral observables are the outcome of the selected decision (the choice), and the associated decision response time (RT). Consequently, the inverse problem in mixture processes–i.e. attributing a specific decision to a specific cognitive process–is ill-posed and hard to solve. Traditionally, in the absence of strong computational *a priori* on how contextual information can be used by the different cognitive systems to converge to a specific decision, a research strategy to dissociate two processes is to postulate that one requires on average less processing time than the other [2]. This entails that, on average, decision RTs from one process are faster than decision RTs from the other process. However, as illustrated recently, RT differences between conditions alone do not suffice to argue for dual processes, since many aspects of the experimental design can influence the mean RT, including differences in perceptual discrimination [12,13] or in preference [8].

For this reason, if one wants to provide support for dual-process theories of cognition, it is crucial to identify other properties in the data that are specific to dual process accounts. Ideally, such properties would never be present in data generated by a single-process.

### Fixed-point property of binary mixture data

**Fig 1:**
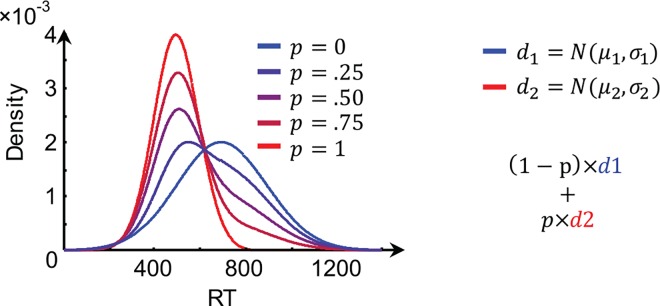
Illustration of the fixed-point property. The blue line reflects an RT distribution for Strategy 1 (d1), with a mean of 700 ms and a standard deviation of 200, the red line a RT distribution (d2) for Strategy 2 (mean = 500, SD = 100). Three additional lines in chromatic variations of purple reflect three mixtures of the two base distributions with mixture proportions as indicated in the legend. Critically, the base and mixture distribution all cross at an indexical *fixed-point*.

One property that meets these criteria is the fixed-point property of binary mixture data [14]. The fixed-point property hold that if two mixtures of distributions share one common density point, then any mixture of those two distributions shares the same common density point (i.e., independent of the mixture proportion, see Fig 1). Therefore, if it is hypothesized that the two processes under consideration contribute to behavior, then an experiment may be divised that manipulates the proportion of experimental trials on which each process contributes. The fixed-point property predicts that in the distributions of the dependent measure (typically RT, but this can be any behavioral or neurophysiological measure) there is a value that occurs equally often in all conditions (Fig 1).

For example, assume that participants are asked to perform a perceptual choice task. Under one instruction, they are asked to choose as fast as possible without concern for the accuracy of their response. In this case, a good strategy would be to randomly guess as soon as the stimulus appears [15]. Under another instruction, participants are asked to carefully choose, making sure that they choose correctly. A good strategy in this case would be to accumulate evidence for each alternative until an accurate decision can be made (e.g., [16]). A simple prediction would be that if participants are instructed to mind both the speed of their response and the accuracy, that the observed behavior would be a mixture of the two response time distributions of the two extremes (i.e., fast guessing and slow accurate choosing). The fixed-point property entails that in such an experiment, the response time distributions would all cross at the same RT. If this property would be found, it would be strong support for two strategies in doing this task (in previous work, we found no evidence for two strategies in exactly this example, [11]).

At this point, it should be noted that while in many cases variation on behavior is expressed as changes in the response time distributions of the various experimental conditions, this is not always the case. For example, two-alternative forces choice tasks are often evaluated on the joint distribution of response time and accuracy (e.g., [17,18]), or (neuro-)physiological measures may have been collected (e.g., [19,20]). A fixed density point is also predicted for these measures under mixture conditions, provided that the data come from a pure mixture of processes (see also Boundary condition 3), and each observation contributes a data point.

The fixed-point property is a particularly useful property of distributions of measured behavior for which two cognitive processes are hypothesized. In particular, because the actual distributions are considered, it is completely model-free with regard to the computations made for the different processes. Thus, a mechanistic theory of the processes is not required for identifying the presence of the processes. In addition, an analysis of the fixed-point property may reveal two processes, even when these processes generate similar behavior. For example, it is not required that one strategy preferentially leads to choosing one option type and the other strategy preferentially leads to choosing the other. Consequently, the fixed-point property analysis does not suffer from the reverse inference problem identified by Krajbich et al. [8].

It is possible to statistically test for the presence or absence of the fixed-point property in experimental data. The procedure for doing so is explained in detail elsewhere [21–23], but entails three steps. In step 1, distributions of behavior for each participant and condition are estimated using a kernel-based density estimator. This means that based on the observed data pattern, a smooth estimate is selected that summarizes how the behavior is distributed. Typically, there is some overlap of the density estimates (see e.g. Fig 1). Therefore, in step 2, for each pair of distributions the point where the density functions cross is computed. Thus if there are three conditions (e.g., the two extremes and one mixture), then each of these pairs of distributions cross at a certain value. This leads to three crossing points because there are three possible pairings of the three experimental conditions (that is, Condition 1—Condition 2, Condition 2—Condition 3, and Condition 1—Condition 3) *per participant*. In step 3, analysis of variance is used to determine whether across participants these crossing points are likely to come from multiple populations–which would be evidence *against* a fixed-point, or whether they are more likely to be drawn from the same population–which would be in favor of a fixed-point. Because traditional null hypothesis significance testing typically does not quantify support for a null hypothesis (but only indicates whether the data is *unlikely* to occur under the null hypothesis), this is often done using a Bayesian statistical analysis [24]. Still, even if the existence of a fixed-point provides strong evidence in favor of a mixture, this property is only a necessary but not sufficient condition for the existence of a mixture process, and one could observe a fixed point in the absence of an underlying mixture of processes. However, it should be noted that the probability of this happening is extremely low.

In the current article, we provide a cautionary note to the application of the above-sketched analysis by identifying three boundary conditions under which the fixed-point property will not be interpretable. In Boundary condition 1, a finding in support of the fixed-point will be mute because of a lack of difference between conditions. Boundary condition 2 refers to the case in which the extreme conditions are so different that a mixture may display bimodality. In this case, a mixture hypothesis is clearly supported, yet the fixed-point may not be found. In Boundary condition 3 the fixed-point may also not be present, yet a mixture might still exist but is occluded due to additional changes in behavior.

## Boundary condition 1: The case of no-effect

The fixed-point formally tests whether the shared density points (that is, the crossing points) of each pair of densities do not differ. That is, if no effect can be detected using an F-test, or if a Bayes factor indicates support for the null hypothesis that there is no effect, then the fixed-point is supported [23].

However, there may be more reasons why a statistical test is in support of the null hypothesis. For example, F-tests may suffer from a lack of power, yielding no significant result when a true difference is present (i.e., a Type II statistical error). The Bayes factor does not suffer from this, and will simply indicate that there is no support for either the null or the alternative hypothesis.

An important case to consider is when the experimental manipulation did not have an effect on all conditions. This may occur for example when the manipulation fails to affect the true underlying mixture proportion of cognitive processes in at least one of the conditions. This may also occur when the manipulation affects the true underlying mixture proportion of cognitive processes in all conditions, but the chosen measure failed to be sensitive to such changes. This might notably happen when experimental designs differ from our typical example, which combines time pressure manipulation and reaction-time measures, by including more subtle manipulations or using a measure with lower signal–to-noise ratio (e.g. response categories, rating scales, or (neuro)physiological measure such as pupil size or electro-encephalograms). In those cases, several conditions could have identical outcomes (cf., identical RT distributions). By necessity, as the complete distribution is identical, so is the common point (or, there is no identified single crossing point). Consequently, a test of the difference in crossing points will not find support for the difference, and ultimately would support the presence of the fixed-point property. Obviously, this inference would be incorrect.

**Fig 2:**
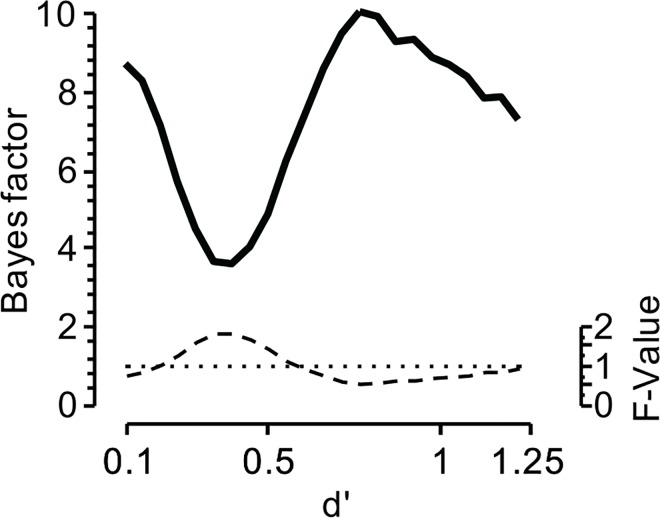
Bayes factors (solid line) as well as F-values (dashed lines) reflecting the presence or absence of the fixed-point property in simulated data. Small effect sizes (indicated by low *d’* values) yield relatively high Bayes factors as well as relatively low F-values, suggesting a fixed-point. The dotted horizontal line indicates the case where there is neither evidence in favor nor against the fixed-point property.

Fig 2. illustrates this boundary condition by presenting mean Bayes factors and F-values for simulated data. The simulated data is from Simulation 2 in [23]. That is, we simulated 50 participants each contributing 200 observations per mixture condition. The mixture proportions were 0, .5, and 1, representing a case in which one condition is a 50/50 mixture of the other two. As Fig 2 shows, the Bayes factor indicating how likely it is that a fixed-point exists in the data, is relatively high for small differences (expressed by low *d’*) between the two base (i.e., unmixed) distributions. Conversely, the F values associated with small differences are low. Accordingly, the conclusion that a fixed-point is present may not be warranted as these Bayes factors and F-values are driven by the low *d’*, rather than a *true* fixed-point.

While a failed operationalization is never a good outcome of an experiment, in the case of the fixed-point property analysis extra care is needed to infer that the experiment was successful, since the primary analysis is on the distribution of crossing points, and not on the observed data. Other mixture analyses [25–29] do not suffer in the same way from this issue, since in those cases the support for a mixture distribution is evident from a property of the data, such as the estimated means of multiple components of the mixture distributions. In the case of no-effect, these means overlap.

To safeguard against the case of no-effect we recommend to first establish that the response times distributions themselves differ *before* performing a test on the crossing points. Since a test on the crossing points is only meaningful if there are at least three condition differences, we recommend an omnibus test followed by post-hoc tests (e.g., pairwise t-tests). If at least three of these post-hoc tests reveal significant differences in the means of the behavioral distributions, then one can proceed with the fixed-point analysis. This test is included in the *fp* package for the statistical programming language R, which can be used to test for the presence of a fixed point in data [23] (The *fp* package can be obtained from http://leendertvanmaanen.com/fixed-point).

## Boundary condition 2: The case of bi-modality

**Fig 3:**
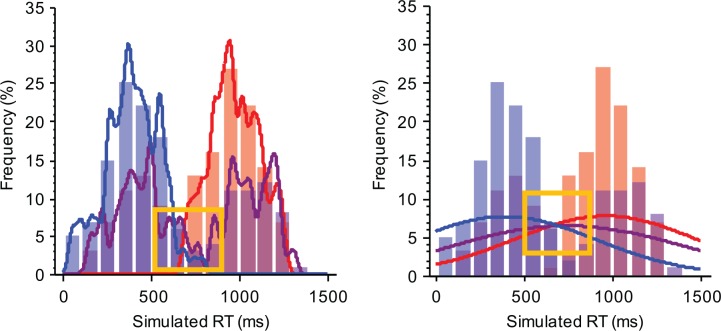
The effect of bandwidth selection on estimating crossing points. Both panels represent three conditions, each consisting of 200 observations. The lines (in blue and red) represent conditions in which one or the other strategy is selected (the base distributions). In this example the samples are drawn from normal distributions with a SD of 150 ms and a mean of 400 ms and 1,000 ms respectively. The purple solid line represents a binary mixture with a mixture proportion of p = .5. The left panel shows a density function with a small kernel bandwidth of 20 ms. In this case, the estimated distribution suggests bimodality. However, the estimated distributions do not display a fixed-point, as evidenced by the lack of a crossing point in the orange square. The right panel shows a density function with a kernel bandwidth of 500 ms. In this case, the fixed-point is retrieved, yet the bimodality is lost.

If the base distributions have very disparate modes, then it may occur that in some conditions the observed (mixture) density is bimodal (Fig 3). In this case, a mixture hypothesis seems highly plausible, since bimodality is a hallmark feature of mixture distributions [10,30]. Yet, the fixed-point property can be more elusive under these circumstances.

This may occur because of sampling error in the data, which may lead to multimodality in a density-based approximation if the smoothing bandwidth is too small. However, a small bandwidth accurately describes the bimodal distribution. Consequently, an accurate description of the data in this case may not yield accurate crossing point estimates. In practice, that means that in order to estimate the crossing points one needs to sacrifice precision in the estimation of the two modes. However, as previously shown by Van Maanen and colleagues [23], if one selects a kernel bandwidth that exceeds one standard deviation of the distributions of all conditions–including the bimodal distribution–then precision in the crossing point estimation is maintained. This is critical, not only to account for potential bimodal distributions, but also in cases where the granularity and potential bounds of the chosen measure—e.g. distributions of ratings on a Likert scale- would not yield a standard, unimodal distribution.

In fact, in the absence of sampling error, any approximation of the data using kernel density estimation with Gaussian kernels yields a fixed-point, independent of the bandwidth *h*. That is, the kernel density function of each base distribution is:

$${\hat{f}}_{i}(x)=\frac{1}{nh}\sum_{j=1}^{n}\varphi (\frac{x-x_{i}}{h})=\frac{1}{h}\varphi \left(\frac{x-x_{i}}{h}\right),$$

with *i* ∈ {1,2} (ie, response 1 or 2), and *φ*(.) the standard normal distribution. Because a mixture of the base distributions is *g*(*x*) = *pf*_1_(*x*) + (1 − *p*)*f*_2_(*x*), and therefore $\hat{g}(x)=p\hat{f_{1}}(x)+(1-p)\hat{f_{2}}(x)$, we can show that there is a fixed density point independent of the mixture proportion *p* in the smoothed density functions:

$$\hat{g}(x_{0})=\hat{f_{1}}(x_{0})=\hat{f_{2}}(x_{0}),$$

$$\hat{g}(x_{0})=\frac{p}{h}\varphi \left(\frac{x_{0}-x_{1}}{h}\right)+\frac{\left(1-p\right)}{h}\varphi \left(\frac{x_{0}-x_{1}}{h}\right),$$

$$\hat{g}(x_{0})=\frac{p+(1-p)}{h}\varphi \left(\frac{x_{0}-x_{1}}{h}\right)=\frac{1}{h}\varphi \left(\frac{x_{0}-x_{1}}{h}\right)∎.$$

Note that this holds for every bandwidth *h*, but that the estimated fixed-point this way differs. If one is only interested in the *presence* of a fixed-point, and not its location, a practical research strategy is to select the bandwith in such a way that the resulting density estimate is smooth. The *fp* R package that can be used to analyse fixed points tests if this is the case. If the densities are such that more than one crossing point exists in any of the condition pairs a warning is provided, as this may be indicative of a lack of precision in the crossing point estimate.

Obviously, one should refrain from interpreting the lack of bimodality in the density functions obtained in this way, since possible additional modes are smoothed out. Additionally, since bimodality is a clear sign of a mixture distribution, there are other and potentially more suited methods to assess the presence of multiple modes. These include clustering algorithms such as mclust [27,28], distributional RT fitting programs in which the number of components can be specified [26], or mode testing software that compute the number of modes in a distribution [29].

## Boundary condition 3: The case of shape change

**Fig 4:**
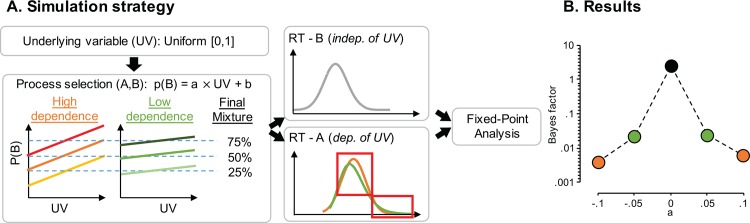
The effect of dependence between RT and mixture probability on estimating crossing points. A. Simulation strategy (see text for details). B. Results. Median Bayes factors for each level of dependence *a*. The simulation illustrates that a Bayes factor supporting the presence of the fixed-point is only found if the dependence between p(B) and UV is (nearly) zero (i.e., *a = 0*).

If the experimental manipulation affects some aspect of the data *in addition* to the mixture proportion then the fixed-point property may not apply. Experimental manipulations might specifically induce 1) a shift of one or both of the base distributions, or 2) a shape change of one or both of the base distributions. Under these circumstances, the fixed-point may not be detectable despite the actual true generative process being a mixture of processes (Fig 4). This is true for any mixture analysis that does not specify the underlying generative processes (cf [25,28]).

Several situations might impair the ability of the fixed-point to correctly detect a mixture of strategies. For example, in the example of a *speed accuracy trade-off* introduced earlier, the fixed-point test would correctly identify a mixture of strategies, only in the case where there is a pure modification of the mixture proportion between a fast-guess and a slow-decision strategy–e.g. the proportion of fast guesses is increased in response to a time-pressure manipulation. If, in addition to the mixture proportion modulation, the level of response caution–i.e. the way response times are generated by the fast-guess and/or slow-decision strategies- is affected (e.g., the level is decreased so RTs are shorter [19,31]), then the fixed-point will not apply. Likewise, the fixed-point will not apply if the mixture probability is not independent of the generated distributions: for example, if the fast-guess process is privileged when the slow-decision computation time would be too long, e.g., when the decision is difficult -this would tend to impact (shrink) the right-tail of the slow-decision RT base distribution, hence induce a shape change which could invalid the fixed-point inference.

To understand the influence of changes in the base distributions on the fixed-point property, we simulated a response time experiment in which the mixture probability depended on the cumulative distribution from one of the base distributions. That is, the probability that a response from one specific sub process is generated increases for slower responses. This reflects for example the case in which the probability of generating a response from sub process B (which for example reflects a heuristic strategy), depends on the difficulty of a choice, reflected in the response time.

Similar to the simulation for Boundary condition 1, we simulated 50 participants that each contributed 200 observations per condition. The means of two inverse Gaussian base distributions were 200 and 400 ms (scale 100 ms and 500 ms, respectively), which yields a large enough effect size that Boundary condition 1 does not apply. The mixture proportions were set at 0.1, 0.5, and 0.9.

The simulation tested for various levels of linear dependence between RT and the probability of being drawn from one base distribution (Fig 4A). Thus the probability of sampling from process B depends on a standard uniformly distributed variable: *p*(*B*) = *a* × UV + *b*. To maintain the preset mixture proportion, we determined the parameters of the regression function in such a way that the function integrates to the mixture proportion *p*: $\int_{0}^{1}(a\times \text{UV}+b)d\text{UV}=p$. The uniform samples are then rescaled according to the regression equation, and used to select whether they generate an RT from base distribution A or B. Crucially, the response times of process B also depend on p(B) using the quantile function of the inverse Gaussian distribution. This leads to a change in the shape of base distribution B (visible in the red squares in Fig 4A), that depends on the probability of sampling from B.

The results of our simulation clearly illustrate the detrimental effect of a RT-dependent mixture probability. If the dependence *a* increases (in the absolute sense), the evidence for of a fixed-point in the data decreases (Fig 4B).

Similar to the simulation, any shape change or shift of one or both of the base distributions can also occur due to misconceptions in the experimental design: for example, this can happen in the case where researchers manipulate the “difficulty” of choices, in order to modulate the proportion of fast-guesses. Then the manipulation may not only change the mixture proportion by increasing fast-guesses, but it may also change the RT base-distribution of the slow decision process (shift to slower RTs due to increasing computation time). Finally, cognitive processes are often subject to learning effects, which might have a significant impact on the modulation of RT base distributions over time.

## Discussion

The fixed-point property of binary mixture data is a strong test of the general hypothesis that two qualitatively different cognitive processes may underlie observed data in a particular domain. In this article we discussed three specific cases in which the fixed-point property may not apply. The first case is the simple situation that an experimental manipulation yielded no effect at all. This may seem trivial, but when the effect is expected to be very small this may be a realistic scenario when analyzing the response time data for a fixed-point. The second case refers to a situation where the data are clearly bimodal, and hence are very likely to be the result of two underlying cognitive processes. In this case, one may be tempted to try and compute the fixed-point using incorrect estimates for the density kernel standard deviations, leading to a decreased probability of finding a joint crossing point. In the third case, even though the data come from two different cognitive processes, the fixed-point may not be found because the experimental manipulation also affected one (or both) of these processes, in addition to the mixture proportion.

A note is warranted on alternative approaches to study dual-process theories. While the model-free analysis of a potential fixed-point is appropriate in the absence of predictive models of the various strategies underlying the (unmixed) behavior, in many cases such models are available. In those cases a rigorous model comparison may be advisable, for example between a model that captures behavior by a mixture of two strategies, versus a model that captures behavior as parametrizations of one strategy [23,32]. Such model-based approaches are not affected by the boundary conditions introduced in the paper (particularly Boundary Condition 3), but building models is more difficult and typically relies on strong theoretical a priori about the computations executed by the two system of interest, and about the arbitration between them [4]. Also, model comparison strategies are not devoid of confound or misinterpretations. That is, a favorable outcome of the comparison for a particular model only indicates that that model should be prefered over the other model. It is not necessarily a good model [33,34]. Thus in case of a winning mixture model over a model that captures behavior as a parametrization of one strategy, the model comparison does not provide support for the mixture model per se. In contrast, this evidence is provided by the fixed-point property. Generally, model-free, data-driven (e.g. fixed-point analysis) and model-based, theory-driven (computational modelling and model-comparison) approaches are complementary and can inform each other. Thus, an interesting analysis strategy to investigate binary mixtures in cognitive sciences could be to inform mechanistic models by first assessing whether a (absence of) mixture of different processes underlies an observed response, using the fixed-point property [35].

One general aspect of the fixed-point property analysis that emerges from the considerations put forth in this article is that it is a priori very unlikely that a true fixed-point is present in the data. Only when the data come from a binary mixture, the experimental manipulation is strong enough to affect the mixture proportions, only the mixture proportions are affected (and no other property of the data), and the analysis is conducted correctly (i.e., an appropriate kernel is selected), only then will it be likely to find a fixed-point in the data. Consequently, even a relative small likelihood that the fixed-point exists in a particular data set should be considered strong evidence, as the prior is strongly *against* finding evidence of dual processes.
